# Mechanisms involved in cancer stem cell resistance in head and neck squamous cell carcinoma

**DOI:** 10.20517/cdr.2022.107

**Published:** 2023-02-21

**Authors:** Juliana Mota Siqueira, Daniele Heguedusch, Camila Oliveira Rodini, Fabio Daumas Nunes, Maria Fernanda Setúbal Destro Rodrigues

**Affiliations:** ^1^Department of Stomatology, Discipline of Oral and Maxillofacial Pathology, School of Dentistry, University of São Paulo, São Paulo 05508-000, Brazil.; ^2^Department of Biological Sciences, Bauru School of Dentistry, University of São Paulo, São Paulo 17012-230, Brazil.; ^3^Biophotonics Applied to Health Sciences, Nove de Julho University, UNINOVE, São Paulo 01504-001, Brazil.

**Keywords:** Head and neck squamous cell carcinoma, cancer stem cell, chemotherapy, radiotherapy, therapy resistance

## Abstract

Despite scientific advances in the Oncology field, cancer remains a leading cause of death worldwide. Molecular and cellular heterogeneity of head and neck squamous cell carcinoma (HNSCC) is a significant contributor to the unpredictability of the clinical response and failure in cancer treatment. Cancer stem cells (CSCs) are recognized as a subpopulation of tumor cells that can drive and maintain tumorigenesis and metastasis, leading to poor prognosis in different types of cancer. CSCs exhibit a high level of plasticity, quickly adapting to the tumor microenvironment changes, and are intrinsically resistant to current chemo and radiotherapies. The mechanisms of CSC-mediated therapy resistance are not fully understood. However, they include different strategies used by CSCs to overcome challenges imposed by treatment, such as activation of DNA repair system, anti-apoptotic mechanisms, acquisition of quiescent state and Epithelial-mesenchymal transition, increased drug efflux capacity, hypoxic environment, protection by the CSC niche, overexpression of stemness related genes, and immune surveillance. Complete elimination of CSCs seems to be the main target for achieving tumor control and improving overall survival for cancer patients. This review will focus on the multi-factorial mechanisms by which CSCs are resistant to radiotherapy and chemotherapy in HNSCC, supporting the use of possible strategies to overcome therapy failure.

## INTRODUCTION

Head and neck malignancies are now the seventh most common type of cancer worldwide^[1]^. More than 90% of head and neck tumors are derived from mucosa epithelium and are diagnosed as squamous cell carcinoma (HNSCC). Although sharing identical histological subtypes, HNSCC can be divided into at least two genetic subclasses based on the absence or participation of human papillomavirus (HPV) in carcinogenesis^[2]^. The oral cavity represents the main subsite for HPV-negative tumors and the oropharynx for HPV-positive ones^[3]^. Moreover, these subgroups also differ in clinical profile, tumor behavior, survival rates, and prognoses^[4]^.

The mainstay treatment for HNSCC consists of surgery with adjuvant or neoadjuvant chemotherapy and radiotherapy. More recently, immunotherapy with checkpoint inhibitors has been indicated for recurrent and metastatic HNSCC with promising results, although only a subset of patients with HNSCC has shown a response to this therapy^[5]^. TNM stage of the disease and anatomic subsites influence therapeutic options for HNSCC. While radical surgeries are the first choice for locally advanced oral cancer, the main treatment for oropharyngeal tumors is chemoradiotherapy, regardless of HPV status. Nowadays, transoral surgeries (robotics and laser microsurgery) have also been performed in the oropharynx region^[6]^.

Despite the advances in current therapy, the prognosis of HNSCC remains poor. More than half of patients die from the disease or complications within a short period, varying from a few months to five years^[7]^. The primary cause of mortality is related to resistance to therapy which leads to local recurrence, cervical lymph node metastasis, and occasionally, distant organ metastasis^[6]^. Tumor heterogeneity and cancer stem cells (CSCs) are known to enhance metastatic dissemination and therapeutic resistance, contributing to lethality^[8]^.

CSCs represent a small but critical subpopulation of cells in the tumor capable of self-renewal and multilineage differentiation and regenerating a tumor when serially transplanted into mice models^[9]^. Since tumors can regrow from a single CSC, cancer treatment success may be attributed to the complete eradication of CSCs populations^[8]^.

Besides, CSCs also demonstrate cellular plasticity; they can reversibly switch between different stem cell phenotypes and between a stem and non-stem cell state^[10]^. CSCs activity is modulated by different signals and cellular interactions provided by the tumor microenvironment, allowing CSCs to achieve highly invasive and aggressive behavior or resist conventional therapies. Thus, activating the Epithelial-to-Mesenchymal Transition (EMT) program by the CSCs represents a valuable strategy to promote invasion, metastasis, and treatment resistance^[10-12]^.

CSCs may originate from adult stem cells or progenitor cells in which the accumulation of mutations over time leads to the activation of transcriptional gene signatures and signaling pathways related to the maintenance of stem cell phenotype and malignant transformation^[13-15]^. Moreover, differentiated cells can also acquire stemness traits due to genetic instability throughout their division process and dedifferentiate, acquiring stem cell properties^[16,17]^. It is essential to highlight that malignant cells can dedifferentiate and acquire stem cell characteristics under challenging situations, including exposure to chemotherapy and radiotherapy^[18]^.

In HNSCC, Prince *et al.* first described the presence of a small fraction of CD44-positive cells capable of generating new tumors when inoculated in immunocompromised mice and re-establishing original tumor heterogeneity^[19]^. Moreover, this subpopulation expressed the *Bmi1* gene, a stemness marker involved in tumorigenesis and self-renewal^[19]^. Since then, other common HNSCC CSC markers, such as CD44, ALDH1, CD133, c-Met, and Bmi-1, have been described^[20-22]^. ALDH1 is considered a highly specific CSC marker, mainly when evaluated with CD44^[20]^. Moreover, based on CD44 and EpCAM expression levels, CSCs in oral squamous cell carcinoma (OSCC) seem to switch between two distinct phenotypes. First, CD44^high^/EpCAM^high^ presents an epithelial morphology and colony formation capability, and second, CD44^high^/EpCAM^low^ has a mesenchymal morphology (EMT profile) with high invasive potential, metastasis and radioresistance ability^[10,23]^. More recently, LIN28A and LIN28B proteins, located in the cytoplasm and nucleus/nucleoli, respectively, were identified as reprogramming factors that can lead to the de-differentiation of malignant oral squamous cancer cells into CSCs and contribute to their immune evasion^[24]^.

For other types of cancers, distinct CSCs can be identified and isolated by fluorescence-activated cell sorting (FACS) using phenotypic surface markers alone or in combination. More than 40 surface markers are known to identify CSCs in solid tumors, and the majority are derived from embryonic or adult stem cells^[25]^. In general, high positivity of CD44, CD24, CD133, CD90, EpCAM, and Aldehyde Dehydrogenase 1 (ALDH1), and elimination of Hoechst 3334 dye *via* ABC transporters are the most used markers^[26]^. The isolated CSCs can be propagated *in vitro* as spheroids or used in organoid cultures. Moreover, spheroid cultures are CSCs enriched, show self-renewal ability *in vitro* and *in vivo,* and generate tumors that resemble the original tumor heterogeneity and differentiation^[27]^.

More recently, in addition to the conventional 2D cell culture, 3D culture models have been used to represent tumor microenvironment heterogeneities properly and reproduce patients’ tumor behavior. Engelmann L. *et al.* developed a 3D Organotypic Co-Culture (3D-OTCs) utilizing HNSCC fresh tissue (non-HPV driven and HPV-driven) placed on top of dermal equivalents (human fibroblasts cultured on a viscose fiber fabric) and analyzed samples’ behavior^[28]^. All non-HPV-driven 3D-OTCs were capable of proliferating cancer cells for up to 21 days and exhibited a heterogeneous, invasive, and expansive growth pattern^[28]^. In the same context, Miserocchi G. *et al.* developed a 3D culture using HPV-positive and HPV-negative HNSCC cells in a collagen-based scaffold. They suggested that the 3D model might induce more mesenchymal phenotypes than 2D cultures^[29]^. Also, in this study, HPV-negative cells presented an upregulation of FLT1 and ABCA3 when seeded in scaffolds, overexpressed EMT-related genes, and increased migration ability compared to HPV-positive cells^[29]^. Based on these findings, collagen-based scaffolds seem to activate drug-resistance mechanisms reassuring the ability of 3D scaffolds to reproduce HNSCC tumor microenvironment impeded by other *in vitro* systems. Accordingly, regarding response to treatment analyses, 3D culture is promising in the future of HNSCC and CSC research.

Several associations between clinicopathological characteristics and CSCs have been appointed in HNSCC, including tumor size, regional and distant metastases, perineural invasion, radiation failure, and poor disease-free survival^[30]^. A previous study of our group explored CSCs markers in tongue tumors and found that the overexpression of CD44 was related to worst overall survival, and Nanog and Oct4 were associated with regional metastasis and death^[31]^. Ma *et al.* suggested that CD133^+^ cells could be responsible for aggressiveness and chemoresistance in oral tumors^[32]^. A meta-analysis study by Fan *et al.* showed that the CSCs markers, CD133, Nanog, and Oct4, could have a prognosis value in HNSCC patients^[33]^. In light of recent events in CSCs markers, there is now some discovery about non-coding RNAs (ncRNAs) used as biomarkers of cancer development and tumor stage determination^[34]^.

MicroRNAs are a type of sncRNA that regulate biological processes. Each miRNA can control target genes and accentuate their potential influence on almost every genetic pathway. Hsieh PL *et al.* demonstrated that ncRNA molecules associated with CSCs are responsible for acquiring and maintaining cancer stemness^[35]^. Let-7 genes family act as a tumor suppressor. Lin28B-let-7 pathway positively regulates the expression of stemness factors Oct4 and Sox2; it causes a switch of non-CSCs to CSCs with tumor starting and self-renewal characteristics in oral CSC^[36]^.

MicroRNA-200 family is another group of genes related to CSC; expression levels of miR-200c were downregulated in ALDH1+/CD44+ HNSCC with BMI1 overexpression. Also, an expression of let-7c or let-7d in oral CSCs suppressed stemness and the radio/chemoresistance hallmarks through suppression of IL-8 or EMT markers, respectively^[37,38]^. MicroRNA-494 acts as a tumor suppressor or oncogenic factor. An increase of miR-494 can inhibit ALDH1 activity, CD133 positivity, and other stemness signatures in ALDH1+CD44+ oral cancer cells. In the same way, activation of miR-494 inactivates Bmi-1 and ADAM10 expression in OSCC-CSCs^[39]^; also, miR-494-3p may enhance the radiosensitivity and induce a senescence pathway in oral cancer cells^[40]^.

In this scenario, it is essential to highlight that CSCs are not easily eliminated by conventional therapies, meaning that after the effective depletion of the bulk of the tumor, residual CSCs populations may survive, drive and sustain cancer recurrence, invasiveness, and therapy resistance^[41]^. Moreover, CSCs are considered intrinsically resistant to chemo and radiotherapy. It is also possible that the CSCs and their close descendants give rise to therapeutic-resistant malignant cells that accumulated mutations caused by genotoxic therapies^[42]^. CSCs adopt different strategies to overcome the challenges imposed by treatment, including the acquisition of dormancy, which is influenced by the CSC niche and immune surveillance, increased drug efflux capacity, activation of DNA repair machinery and decreased activation of apoptosis^[43]^. This review will focus on the mechanisms that lead to CSC resistance to radiotherapy and chemotherapy in HNSCC.

## RADIORESISTANCE AND CSC

In HNSCC patients, radiotherapy (RDT) is a common choice of treatment to achieve cancer control after surgery and/or current chemotherapy^[6]^. Usually, on weekdays patients receive a dose of 70 Gy that can be administered through standard fractionation (2 Gy, once a day) or via accelerated fractionation and hyperfractionation (twice a day)^[44]^. Fractionation guarantees that cancer cells will eventually be exposed to radiation in all cell cycle phases, favoring DNA damage and cell fate. Nevertheless, this process also activates important protein regulators of DNA damage response, such as ataxia-telangiectasia mutated (ATM) and ataxia-telangiectasia and Rad3-related protein (ATR), which will be decisive in treatment response^[45]^.

Tumor response or failure to ionizing radiation is mainly associated with the classical 4 R’s of radiobiology: repair of sublethal DNA damage, reassortment of cells in the cell cycle, cell repopulation, and reoxygenation of hypoxic areas^[46]^. Efficient cell death by RDT depends on producing unrepairable damage involving DNA double-strand breaks (DSBs); however, most radiation-induced DNA damage is sublethal. DNA repair systems include base excision repair (BER), nucleotide excision repair (NER), homologous recombination (HR), non-homologous end joining (NHEJ), and mismatch repair (MMR) pathways^[47]^. In this context, CSCs seem to hold elevated levels of proteins responsible for NHEJ and HR and an increased DSB repair capacity^[23]^.

If tumor recurrences occur within six months following radiation, tumors are considered radioresistant^[48]^. Mechanisms involved in radioresistance are not fully understood, but accumulated evidence indicates that cancer stem cells (CSCs) are decisive in this process^[46,49]^. In general, therapeutic resistance refers to the ability of cancer cells to recover and repair DNA damage and regrow after tumor therapy^[50]^, being higher in CSCs than non-CSC^[51]^. This ability is mainly related to the increased regulation of DNA repair genes, DNA-damage checkpoints, and anti-apoptotic proteins^[52,53]^.

Furthermore, it has been recognized that a CSC subpopulation exhibiting a mesenchymal profile (CD44^high^/CD24^low^) presents an even higher level of DNA repair following RDT^[23]^. Besides, irradiation activates stemness pathways and induces CSC phenotypes in non-stem cancer cells. Up-regulation of CSCs genes, such as Sox2 and Oct3/4, may be observed after radiation, contributing to tumor radioresistance^[53]^. The plasticity of CSCs dramatically interferes with identifying and eliminating CSCs during cancer therapies^[54]^.

Radiation promotes an arrest of CSCs in the G2/M phase, which allows active DNA repair. Moreover, after radiation, there is a noticeable discrepancy between the higher rates of self-renewal and proliferative abilities of CSCs compared to their lower apoptosis activation, favoring tumor growth^[52]^. In oral cancer cell lines, changes in CSCs content (ALDH+) are associated with an increase in the rates of sub-lethal damage repair (SLDR), which enables efficient cell repair and reduces tumor control capabilities^[55]^. Duration of the exposure to the fractionated dose-delivery of radiation seems to influence radioresistance mechanisms driven by SLDR, suggesting that reduced overall dose-delivery time on radiotherapy could favor CSCs control^[55]^.

Besides the DNA repair process, activation of checkpoint responses after radiation damage also participates in the radioresistance of several tumors, including HNSCC. Cell cycle progression is delayed to allow DNA repair through activation of signaling pathways such as ataxia telangiectasia mutated (ATM)-checkpoint kinase 2 (Chk2) and ATM-Rad3-related (ATR)- checkpoint kinase (Chk1)^[56]^. CSCs appear to enhance response to DNA damage activating Chk2 in invasive oral cancer^[23]^. Inhibition of Chk1 was suggested as a therapeutic target in HNSCC that contributes to the failure of DNA replication and intensification of DNA damage^[57]^.

Induction of apoptosis represents one of the primary mechanisms by which cancer cells are eliminated in cancer therapies^[58]^. Reduced cleaved caspase proteins showed the apoptotic resistance of CSCs in oral cancer after irradiation^[23]^. Resistance mechanisms evolving upregulation of anti-apoptotic proteins such as Bcl-2 and inhibitor of apoptosis (IAP) are commonly found in tumor cells, especially in CSCs^[59]^. Radiation can activate X-linked IAP (XIAP), another IAP family member that inhibits apoptosis mediated by mitochondrial and caspase-3 pathways^[60]^. Besides apoptosis regulation, Bcl-2 family members also participate in cell migration, invasion, and metastasis^[61]^. In this focus, an inhibitor of Bcl-2 combined with Cetuximab and radiation showed excellent results in eliminating CSCs in HNSCC cell lines^[62]^.

Another widely studied mechanism of CSCs contributing to radioresistance and poor prognosis in HNSCC is related to hypoxia, i.e., low oxygen levels caused by insufficient blood supply to tumor tissues^[63,64]^. A hypoxic tumor environment can interfere directly with the potential of radiation to damage DNA cells and indirectly regulate the expression of genes related to aggressiveness and response to treatment. Additionally, hypoxia is essential in protecting the CSCs niche from radiation effects and in acquiring and maintaining CSC-like phenotype^[65]^.

In HNSCC, hypoxia-inducible factor-1a (HIF-1α), a transcriptional regulator of oxygen homeostasis, is enhanced in CSCs subpopulations in response to radiation^[66]^. Furthermore, hypoxia upregulates CSCs genes such as *Sox2* and *Nanog*, consequently contributing to the survival of tumor cells after radiation^[67]^. Linge *et al.* showed a correlation between high tumor recurrence after postoperative radiochemotherapy in locally advanced HNSCC patients, increased expression of CSCs markers, and high hypoxia-induced gene signature expression^[68]^. Strategies for hypoxic modifications such as hyperbaric oxygenation or nitroimidazoles significantly reduced locoregional recurrence after radiation in HNSCC^[69]^.

In the same context, reactive oxygen species (ROS) and redox-regulatory mechanisms can regulate DNA damage and resistance to irradiation. Accumulation of ROS and DNA damage of cancer cells is associated with the effectiveness of radiotherapy^[70]^. Unlike non-CSCs, CSCs present a high antioxidant capacity that coordinates the activity of free-radical scavengers and protects cells from induced-radiation death^[70,71]^*. *This low ROS state presented by CSCs is also related to the quiescent state of HNSCC stem cells and enhanced tumorigenic potentials *in vitro* and *in vivo*^[72]^. Interestingly, GDF15 (growth differentiation factor 15), a member of the TGF-β superfamily, participates in ROS suppression in HNSCC, contributing to radioresistance and acquisition of the CSC phenotype^[73]^. Boivin *et al.* showed that redox-modulating by inhibiting GSH antioxidant system previous to radiation is an accurate strategy to eliminate highly tumourigenic CSCs^[74]^.

Considering the better prognosis of HNSCC HPV-positive patients, it seems that HPV may influence several molecular mechanisms involved in CSC’s radiosensitivity^[75,76]^. Rieckmann *et al.* demonstrated a limited capacity of DSB repair in HPV/p16-positive cancer cells^[77]^. HPV-positive tumors are believed to present less radioresistant CSCs subpopulations due to their reduced repopulation ability during radiation therapy^[78]^. Reid *et al.* explored irradiation behavioral responses of CSCs with CD44^+^ ALDH^+^ phenotype in 6 HPV positive and negative HNSCC cell lines^[79]^. Their principal findings showed that HPV status did not influence the inherent proportions of CSCs, which were changed in both groups in response to radiation. HPV-negative samples showed a significant increase in CSCs densities, probably reflecting their remarkable repopulating ability after treatment^[79]^. Other studies demonstrated that HPV-negative cell lines seem more capable of dedifferentiating from non-CSCs to CSCs in response to radiation than HPV-positive cell lines^[80]^. In addition, low levels of functional TP53 expressed by HPV-positive cells may contribute to inducing apoptosis following radiotherapy^[81]^.

In an attempt to address this issue, the literature has found that cisplatin-sensitization has helped overcome resistance to radiation in many patients. In a recent study, Routila *et al.* appointed *Oct4* as a good marker for identifying radioresistance and cisplatin-sensitive tumors, which could help distinguish patients who should receive cisplatin-sensitization from those who would not benefit from this therapy^[82]^. In summary, Oct4 positivity reduced cancer cell apoptosis, favoring cell viability after irradiation. At the same time, Oct4 can contribute to cisplatin mechanisms inhibiting DNA repair activation^[82]^. In radioresistance, Oct4 driving activates the oncogene Cancerous Inhibitor of Protein Phosphatase 2A (CIP2A), which promotes malignant cell growth and proliferation^[50]^.

**Figure 1:**
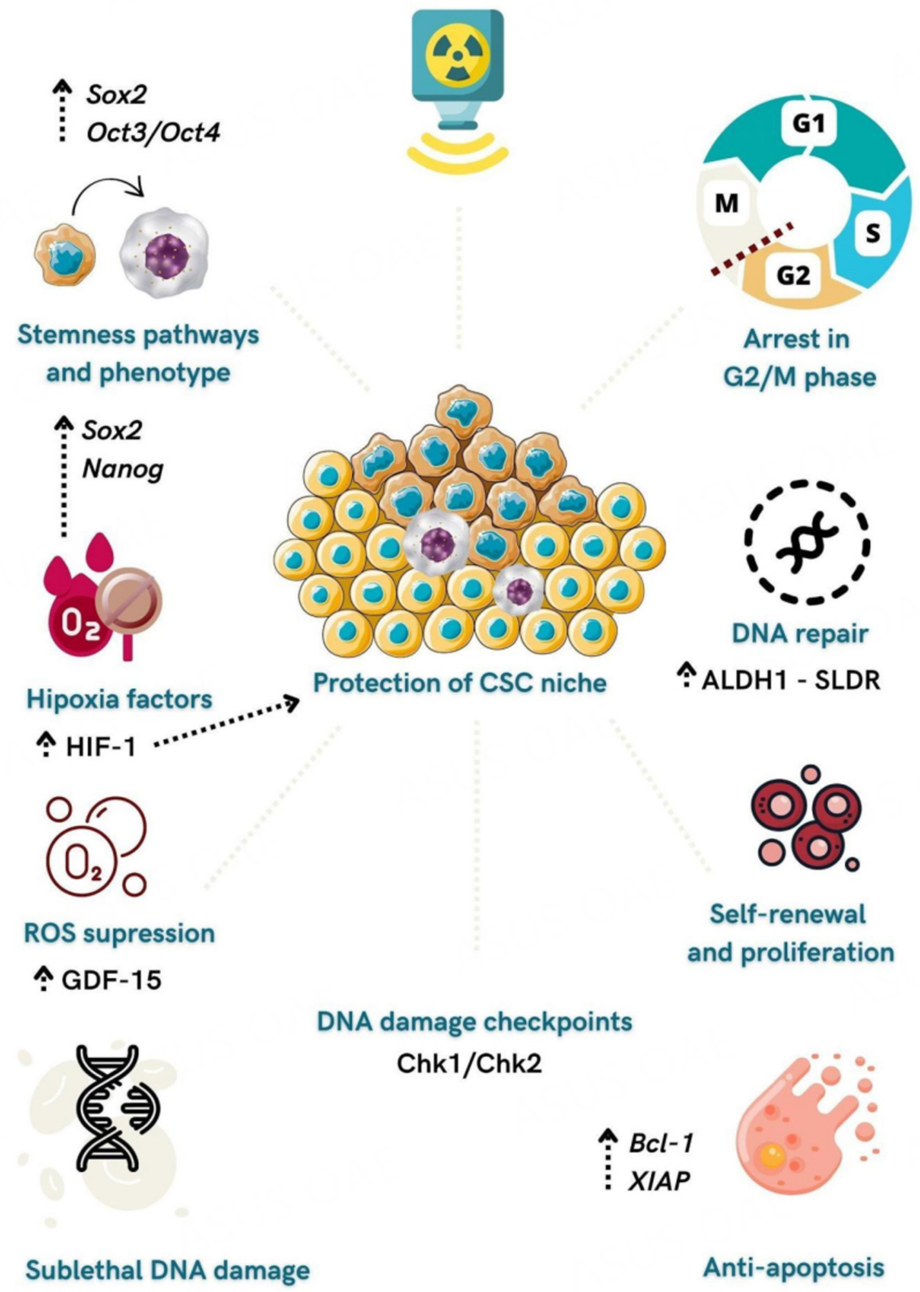
Mechanisms related to CSCs radioresistance in HNSCC. Radiation can activate stemness pathways such as Sox-2 and Oct3/4 and induce CSC phenotype in non-stem cancer cells. Radiation promotes an arrest of CSCs in the G2/M phase and activates Chk2 and Chk1, which delays cell cycle progression and allows DNA repair. Overexpression of CSC marker ALDH1 leads to increased rates of sub-lethal damage repair (SLDR), enabling efficient cell repair and reducing tumor control capabilities. CSCs upregulate anti-apoptotic proteins such as Bcl-2 and X-linked inhibitors of apoptosis (XIAP). Hypoxia upregulates CSCs genes (*Sox2* and *Nanog*) and is essential in protecting the CSCs niche from radiation effects. GDF15 (growth differentiation factor 15) participates in ROS suppression in HNSCC, contributing to radioresistance and acquisition of the CSC phenotype.

Despite technological developments, RDT still promotes long-term toxicities compromising the quality of life and is often associated with potential tumor resistance^[6,83]^. CSCs act as key players in regulating different mechanisms of DNA damage repair and other regulators of cell death after irradiation, such as hypoxia, apoptosis, and ROS [Figure 1]. At this point, we believe that RDT is insufficient to eliminate CSCs in HNSCC, explaining the high recurrence rates of these tumors. Thus, further investigation is required to comprehend and overcome CSC’s radioresistance and improve treatment success and overall survival in cancer patients.

## CHEMOTHERAPY RESISTANCE AND CSC

HNSCC in stage I or II (early tumors) is curable with higher survival rates after surgery or radiotherapy alone. In contrast, over 60% of stage III or IV HNSCC (locoregionally advanced) require advanced therapeutic options such as surgery followed by radiotherapy with or without chemotherapy^[7]^. Currently, the standard chemotherapy regimens for stage III or IV, as well as recurrent and metastatic HNSCC, are based on cisplatin, 5-fluorouracil (5-FU), and docetaxel/paclitaxel^[84-86]^.

Chemotherapeutic drugs exert different biological effects on tumor cells, relying on specific mechanisms of action. Cisplatin is a platinum-based alkylating agent that creates inter- or intra-strand cross-links or transfers alkyl groups to the guanine residues of DNA, generating mispairing formation in DNA bases and avoiding strand separation during DNA synthesis^[87]^. On the other hand, 5-FU is a pyrimidine antagonist antimetabolite that interferes with essential biosynthetic pathways, disturbs the DNA/RNA synthesis, or causes the formation of DNA strand breaks through inhibition of particular enzymes or incorporation of false structural analogs of pyrimidine/purine into DNA^[88]^. Docetaxel is a topoisomerase II inhibitor that impairs DNA replication and causes DNA strand breaks. Paclitaxel is a taxane that modifies the function/formation of spindle microtubules by inhibition of nuclear division (mitotic arrest in metaphase), leading to cell death^[87]^. In this context, it is essential to highlight that most chemotherapeutics’ success relies on the drugs’ ability to decrease tumor size or induce short-term remission. This measure of success is intuitive, and many medications evaluated by these criteria are used in effective chemotherapeutic regimens^[89]^.

Although the chemotherapeutic scenario seems broad, mortality from HNSCC continues to rise worldwide^[90]^. As reviewed by Bukowski *et al.*, part of this problem may be a reflection of drug resistance, which leads to a reduction of the therapeutic efficacy and is related to over 90% mortality of cancer patients^[91]^. Multi-drug resistance (MDR) of cancer cells during chemotherapy can be associated with a variety of mechanisms, including enhanced efflux of drugs, drug activation or inactivation, genetic factors (gene mutations, amplifications, and epigenetic alterations), growth factors, increased DNA repair capacity, inactivation of apoptosis machinery, increased autophagy, and elevated metabolism of xenobiotics, or even any combination of these mechanisms^[91-93]^. In addition, establishing a tumor microenvironment (TME) promotes tumor progression and chemoresistance through a collection of soluble proteins and insoluble vesicles secreted by tumor cells. This cell-to-cell communication among various cell types required to form the TME, such as mesenchymal stromal cells, immune cells, and vascular endothelial cells, influences the function of cells in the TME, shapes the premetastatic niche, and is an essential contributor to the development of chemoresistance^[94]^.

Tumor heterogeneity is a significant complicating factor in cancer treatment and is also strictly associated with chemotherapy resistance, impacting poor prognosis for HNSCC patients^[95]^. Specifically, the presence of the CSCs has been associated with resistance to chemotherapeutic agents such as cisplatin, bortezomib, etoposide, 5-FU, and doxorubicin^[92,96]^. Most importantly, many studies have demonstrated that treatment with these drugs enhances the CSCs fraction in different solid tumors and favors EMT traits, leading to treatment resistance and cancer progression^[97,98]^. In addition, the acquisition of resistance to a specific drug generally tends to multiply resistance to unrelated compounds in CSCs and malignant cells, which under treatment pressure, can acquire a stem-like phenotype and become therapeutic resistant^[18]^.

**Figure 2:**
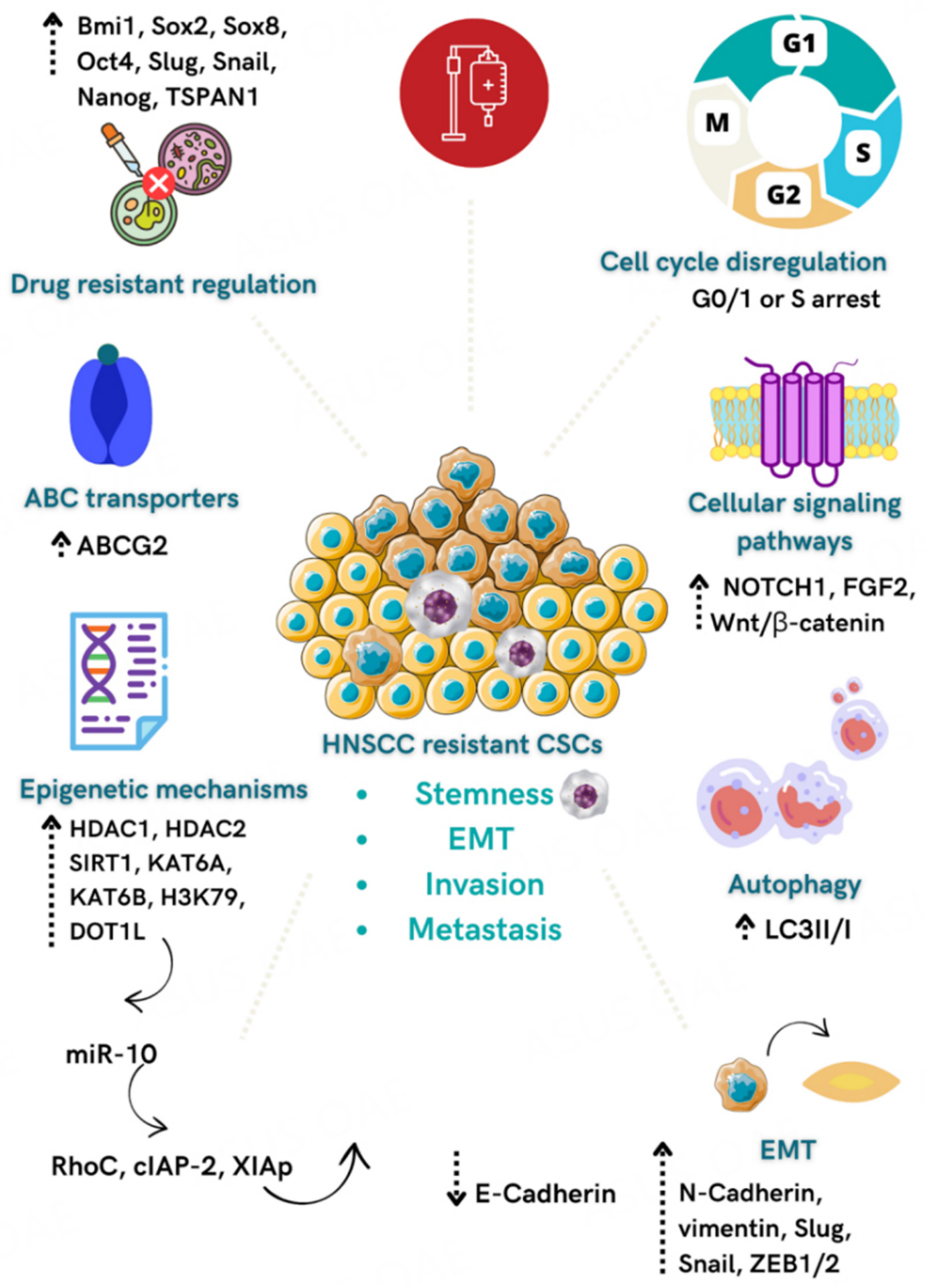
Mechanisms related to CSCs’ chemoresistance in HNSCC. Overexpression of *Bmi1*, *Sox2*, *Sox8*, *Oct4*, *Slug*, *Snail*, *Nanog*, and *TSPAN1* genes leads to the acquisition of drug resistance and stemness, EMT, and metastasis. CSCs activate signaling pathways such as the NOTCH1, FGF2, and Wnt/β-catenin to promote chemoresistance and stemness. Increased expression of ABC transporters, mainly ABCG2, the activation of EMT, cell cycle deregulation, increased autophagy, and activation of epigenetic mechanisms, such as up-regulation of miR-10, are involved with CSC’s chemoresistance in HNSCC.

CSCs were identified as crucial players in the acquisition of drug resistance and unresponsiveness to current chemotherapies against cancer by activating different cellular signaling pathways and mechanisms [Figure 2]. The main reasons found in the literature rely on intrinsic properties of CSCs, such as the (1) inherent quiescent state that enables them to evade the actions of drugs that target rapidly proliferating cells; (2) high levels of drug efflux pumps and detoxifying enzymes; (3) increased DNA self-repair capacity; (4) specific expression of anti-apoptotic and prosurvival proteins; (5) acquisition of the EMT-phenotype; (6) oxidative modulation; (7) epigenetic modifications and (6) activation of the specific signaling pathways^[90,99-103]^. In addition, the role of the TME in sustaining the CSCs niche is also gaining substantial importance in promoting resistance to chemotherapy as an extrinsic factor^[101]^. The TME shapes the morphology and functional features of CSCs, mainly influencing (1) cellular plasticity; (2) hypoxia; (3) metabolic reprogramming; (4) activation of specific signaling pathways; and (5) cell-to-cell interactions^[100]^.

Several *in vitro* studies found that stemness-related genes are overexpressed in HNSCC cell lines resistant mainly to Cisplatin, 5-FU, doxorubicin, and docetaxel. *Sox2*, *Oct4*, *CD44*, *Bmi1*, *ALDH1*, and *Nanog* were the genes most frequently associated with the CSC phenotype. Also, EMT markers (*Slug*, *ZEB1*, *Twist*, *Snail*), as well as drug efflux transporters (ABCG2, ABCC1/ABCC2/ABCC3/ABCC4/ABCC5, ABCB1), epigenetic alterations (HDAC1/HDAC2, SIRT1, KAT6A,/KAT6B), and specific signaling pathways such as Wnt/β-catenin and NOTCH1. These mechanisms endow CSCs to survive against standard cancer therapies and promote tumorigenesis, recurrence, and metastasis after chemotherapy Table 1^[99,104-107]^.

**Table 1:** The main mechanisms involved in chemotherapy resistance of cancer stem cells in HNSCC

**Author**	**Type of study**	**Drug and concentration**	**Cell line**	**CSC isolation**	**Associated genes**	**Main findings**
Oliveira *et al.*^[96]^	*In vitro*	Cisplatin (9-92 µM)	CAL-27 CisR and SCC-9CRR	ALDH1^+^ CD44^+^	*HDAC1,HDAC2, SIRT1, KAT6A, KAT6B, ZEB1, Bmi1*	● The mRNA levels of *HDAC1*, *HDAC2*, *SIRT1*, *KAT6A*, and *KAT6B* were up-regulated in cisplatin-resistant cell lines, indicating activation of epigenetic mechanisms for chemoresistance acquisition ● Activation of EMT program via association of epigenetic regulators and *ZEB1* is involved with resistance to cisplatin ● CSC subpopulation increased in cell lines with increasing levels of cisplatin resistance, which was also associated with high expression of Bmi1
Lee *et al.*^[109]^	*In vitro, in vivo*	Cisplatin (5- 50 µM)	SNU1041 and FaDu	ALDH1^high ^CD44^+^	*Oct4, Sox2* *Nanog, Twist, Snail, Slug*	● *SOX2* overexpression is associated with recurrence and contributes significantly to acquiring stem cell traits in HNSCC cell lines ● SOX2 expression is high in ALDH1^high^ CD44^+^cells, and its down-regulation was followed by *Oct4 *and *Nanog* down-regulation, decrease in stemness, invasion, EMT, and frequency of CD44^+^ cells ● SOX2 contributes to the resistance of CSCs to cisplatin, and its inhibition decreases CSCs viability, possibly by the inhibition of ABCG2. ● Downregulation of ABCG2 in CSCs overexpressing SOX2 restored drug sensitivity after cisplatin treatment
Xie *et al.*^[110]^	*In vitro, in vivo*	Cisplatin (1-10 µM)	SCC9-res cells CAL27-res	CD44^+^ CD24^-^	*Oct4*, *Sox2*, *Bmi1*, *SOX8*, *ABCG2*	● Cisplatin-resistant HNSCC cell lines acquire CSCs properties, characterized by increased* Oct4, Sox2, Bmi1, and ABCG2* expression, self-renewal potential, EMT activation, and tumorigenesis *in vivo*, which was mediated by *SOX8* upregulation ● *SOX8* knockdown decreases the expression of CSCs associated genes as well as *ABCG2* and inhibits sphere formation, CD44^+^ CD24^-^ fraction, migration, and invasion in cisplatin-resistant cell lines ● *EMT *was successfully reversed after *SOX8* knockdown and inhibited metastasis ● Moreover, *SOX8* knockdown repressed tumor metastasis mainly due to inhibition of the Wnt/ β-catenin signaling pathway through the transcriptional regulation of *FZD7*
Nör *et al.*^[111]^	*In vitro, in vivo*	Cisplatin (different concentrations)	UM-SCC-1, UM-SCC-22A, and UM-SCC-22B	ALDH^high^ CD44^high^	*Bmi1*, *Oct4*	● Exposure to 2μM cisplatin for 24h showed no impact on cell survival in malignant cells. However, when sorted ALDH^high^CD44^high^ cells were exposed, cisplatin doubled the CSCs fraction ● Low concentrations of cisplatin-induced the expression of *Bmi1* and *Oct4* genes, CD44, and orosphere formation in unsorted and sorted CSCs, indicating that this therapy contributes to the acquisition and maintenance of stemness
Chen *et al.*^[112]^	*In vivo*	Cisplatin (1mg/Kg body weight)	SCC1, SCC1R, SCC9, SCC22B, SCC23, SCC23R, HN13	Bmi1^+^ EpCAM^+^ (primary mouse HNSCC) ALDH^high^ CD44^+^ EpCAM^+ ^(Primary human HNSCC)	*-*	● Bmi1 identifies a population of CSCs responsible for HNSCC initiation, progression, and metastasis using an elegant *in vivo* model of genetic lineage tracing Bmi1^+^ CSCs are located in lymph nodes and in the invasive front of HNSCC, mediating invasive behavior and metastasis ● Bmi1^+^ CSCs are enriched after *in vivo* treatment with cisplatin and were able to reconstitute the tumor heterogeneity after therapy, indicating that these cells are one of the major causes of recurrence ● Targeting Bmi1^+^ CSCs with Bmi1 or AP-1 inhibitors and the tumor bulk with cisplatin resulted in improved therapeutic outcomes, reduced tumor size, and the incidence of lymph node metastasis *in vivo*
Kulsum *et al.*^[113]^	*In vitro, in vivo*	Cisplatin (2-32 µM), Docetaxel (2-15nM) and 5-FU (5-100µM)	Hep-2, Hep-2 CisR, Cal-27, Cal-27 CisR, Cal-27 5FUR, Cal-27 Dox	CD44^+^, CD133^+^, ALDH1A1^+^	*Oct4, Sox2, Nanog, CD44, NOTCH1, CD133, ALDH1A1, ABCG2*	● Cell lines resistant to cisplatin and 5-FU showed enrichment of CD44^+^, CD133^+^ and ALDH1A1^+^ CSCs, increased expression of *ABCG2*, *Sox2*, *Nanog*, *Oct4,* and *NOTCH1* genes, and cell cycle dysregulation, characterized by G0/G1 or S phase arrest ● Increased spheroid formation and migration were also observed in resistant cell lines ● *Oct4*, *Sox2,* and *Nanog* expression represent driving forces behind the induction of drug-induced chemoresistance in HNSCC ● Depletion of ALDH1A1 with small molecule inhibitor (NCT-501) in resistant cell lines inhibited tumor burden *in vivo* and increased the efficacy of cisplatin in patient-derived *ex vivo* explant
Bourguignon *et al.*^[114]^	*In vitro*	Cisplatin (different concentrations)	HSC-3	ALDH^high^ CD44^high^	*Oct4, Sox2, Nanog*	● HA (matrix hyaluronan) promotes aggressiveness in highly tumorigenic ALDH^high^ CD44^high^ tumor cells ● Up-regulation of *DOT1L* and monomethyl l-H3K79 lead to miR-10 production in HA-treated CSCs ● miR-10 increases the cytoskeleton regulator RhoC in CSCs and *DOTL1* signaling inhibition via DOT1L siRNA or anti-miR-10b inhibitor decreases RhoC, tumor cell migration/invasion, expression of survival proteins (cIAP-2 and XIAP) and contributes to increasing chemosensitivity ● Inhibition of cIAP-2 or XIAP expression enhances cisplatin-induced chemosensitivity in ALDH^high^ CD44^high^ CSCs ● Taken together, *DOTL1 *and miR-10 are important targets for future therapies to decrease stemness, induce CSCs death and increase its susceptibility to standard chemotherapy
McDermott *et al.*^[115]^	*In vitro, in vivo*	Cisplatin (2 μM)	UM-SCC-1 and UM-SCC-22B	ALDH^high ^CD44^high^	*-*	● *FGF2 *and *EREG* mRNA were increased in cisplatin-treated ALDH^high^ CD44^high^ ● TNFα, IFNγ, IL-6, and NF-κB signaling pathways were associated with cisplatin resistance in ALDH^high^ CD44^high ^cells ● FGFR1-4 inhibition, together with cisplatin treatment, promoted a 50% reduction in ALDH^high^ CD44^high^ ● After *FGFR2* knockdown, cisplatin no longer increased the ALDH^high^ CD44^high^ CSC in HNSCC cell lines ● Therapeutic inhibition of FGFR might contribute to eliminating ALDH^high^ CD44^high^ cisplatin-resistant CSCs
Elkashty *et al.*^[117]^	*In vitro, in vivo*	Cisplatin (0.817 µg/mL) 5-FU (3.644 µg/mL)	SCC12 and SCC38	CD44^+^CD271^+^	*Oct4, Sox2*	● CD44^+^ CD271^+^ cells showed increased resistance to oxidative stress in HNSCC (which is a cytotoxic effect of cisplatin) and higher expression of *Bmi1*, *Oct4, Sox2*,* SMO*,* a*nd *GLI1 *genes after exposure to cisplatin and 5-FU
Yu *et al.*^[119]^	*In vitro, in vivo*	Cisplatin (6.25-100 µM), 5-FU (6.25-100 µM) and doxorubicin (1.25-20 µM)	OECM1-SP SCC25-SP	Side Population (SP)	*CD133, ABCG2, ALDH1A1*	● CD133 was significantly up-regulated in SP cells, which also demonstrated high chemoresistance and expression of ABCG2 ● Depletion of CD133 was associated with decreased SP frequency and attenuated *in vivo* tumor formation ● Targeting CD133 together with cisplatin treatment abrogated the proliferation of SP cells in HNSCC, indicating that CD133 is a promising therapeutic target to overcome drug resistance in CSCs
Moon *et al.*^[120]^	*In vitro, in vivo*	Cisplatin (5-100 µM)	YD8, SNU1041, KU-SCC1 and KU-SCC3	CD44^+^	*Slug*	● CD44+ cells showed high expression of Slug and were significantly resistant to cisplatin, which was also associated with an elevated expression of ABC transporters
Koo *et al.*^[122]^	*In vitro, in vivo*	Cisplatin (5-50 µM)	HNSCC cell lines (FaDu, SNU1041, SNU1076, YD15, SCC25, and HN6) and three HNSCC CSCs cell lines (K3, K4, and K5)	*Oct4 *overexpression	*SOX2* *Nanog*	● Oct4 overexpressing cells in differentiated HNSCC cell lines can drive the acquisition of stem-like phenotype ● Oct4 overexpressing cells were more resistant to cisplatin, which was associated with increased expression of ABCC6, indicating that Oct4 is involved in drug resistance
Ota *et al.*^[126]^	*In vitro, in vivo*	Cisplatin (1µM)	SAS and HSC-4	*Snail *overexpression	*Oct4, Sox2, Nanog, Bmi1, ABCG2*	● *Snail* overexpression led to increased expression levels of CD44 and ALDH1 as well as in the expression of *Bmi1*, *Nanog*, *Oct4*, *Sox2, *and *ABCG2* genes ● EMT was induced in *Snail* overexpressing cells, which was also associated with increased stemness and enhancement of chemoresistance ● *in vivo, Snail* overexpression induced an invasive phenotype in non-invasive SAS and HSC-4 cells
Garcia-Mayea *et al.*^[135]^	*In vitro*	Cisplatin and 5-FU (IC50 or higher concentrations)	HTB-43, CCL-138, and JHU029 and their respective cisplatin-resistant cell lines, SCC25	Growing cells in non-adherent conditions for 3 generations	*Sox2, CD44, ALDH1A1, KLF4, ABCB1, Twist*	● CSCs derived from spheres were more resistant to cisplatin and 5-FU when compared with the parental cells ● Cells with higher resistance to cisplatin showed a higher percentage of CSCs ● CSCs demonstrated higher levels of LC3II/I, indicating that autophagy may be involved with CSCs resistance to cisplatin
Garcia-Mayea *et al.*^[136]^	*In vitro, in vivo*	Cisplatin (0-150 μM) Dasatinib (0-3 μM)	HTB-43, CCL-138 and JHU029 and their respective cisplatin-resistant cell lines	Growing cells in non-adherent conditions for 3 generations	*TSPAN1*	● CSCs and cisplatin resistant HNSCC overexpress the *TSPAN1 *gene and protein ● *in vitro*, *TSPAN1* inhibition decreased autophagy and EMT traits, induced apoptosis, increased sensibility to chemotherapy and inhibited the pSrc-signaling cascade ● *in vivo, TSPAN1* depletion impaired tumor growth and metastasis spreading
Mir *et al.*^[138]^	*In vitro, in vivo*	Cisplatin (0-150 μM), Desatinib (5-100nM)	Fadu, CCL-138, CCL-138 CisR, JHU-027, SCC-25, HTB-43	Growing cells in non-adherent conditions for 3 generations	*SDCBP*	● Cisplatin resistant cells and CSCs showed high *SDCBP* levels and formed slow-growing but highly aggressive tumors *in vivo* ● *SDCBP* inhibition promoted cisplatin sensitization in HNSCC cell lines with high resistance to cisplatin, reduced tumorsphere formation, EMT traits, and CSCs fraction identified as SP ● p-Src was identified as a major downstream target in SDCBP-mediated CSC properties and cisplatin resistance in HNSCC ● SDCBP protein expression in HNSCC was associated with advanced tumor stage, shorter disease-free survival and overall survival
Lee *et al.*^[139]^	*In vitro, in vivo*	Cisplatin (5-50 µM)	SNU-1041, FaDu, HNSCC CSCs cell lines (K1 and K3)	-	*ABCC1, ABCC2, ABCC3, ABCC4, ABCC5, nuclear β-catenin target genes ( cyclin D1, cyclin A, Cyclin E and c-Myc)* *Oct4, Sox2, Nanog, CD44, ABCB1, ABCG2* *Wnt 3a, Wnt 5a Wnt 7a, Wnt 10a Wnt 10b, Wnt 13* *FZD2, FZD4* *FZD5*	● Wnt/β-catenin signaling pathway is activated in CSCs cell lines and β-catenin overexpression drives the acquisition of CSCs properties as self-renewal, stem cell marker expression, including *Oct4*, and chemoresistance ● β-catenin directly regulates *Oct4* transcription in CSCs and *Oct4 *overexpression abrogates the inhibition of stemness caused by β-catenin knockdown in CSCs ● Wnt/β-catenin axis mediates the self-renewal of CSCs in HNSCC ● Novel therapeutic strategies for targeting CSCs in HNSCC may focus on the blockade of the Wnt/β-catenin signaling pathway
Byun *et al.*^[140]^	*In vitro, in vivo*	Cisplatin (different concentrations)	SCC-15, SCC-25, fresh HNSCC	CD44^+^	*-*	● CD44^+^ cells were more resistant to chemo and radiotherapy than CD44^-^ cells *in vitro* and *in vivo* ● *in vivo* treatment with cisplatin and radiation increased tumor hypoxia, HIF-1α and the fraction of CD44^+^ cells ● HIF-1α promotes stemness via upregulation of NOTCH1 in HNSCC ● HIF-1α or NOTCH1 knockdown increases susceptibility to cisplatin and radiation, which was mediated by Blc-2 inhibition and caspase-3 expression ● Blocking HIF-1α associated with cisplatin substantially decreased tumor growth *in vivo* ● HIF-1α/NOTCH1 signaling in CSCs can be targeted to impair tumor growth and progression as well as to overcome therapeutic resistance

ABCB1: ATP binding cassette subfamily B member 1; ABCC1: ATP binding cassette subfamily C member 1; ABCC2: ATP-binding cassette sub-family C member 2; ABCC3: ATP binding cassette subfamily C member 3; ABCC4: ATP-binding cassette sub-family C member 4; ABCC5: ATP-binding cassette sub-family C member 5; ABCG2: ATP-binding cassette super-family G member 2; ALDH1A: Aldehyde dehydrogenase 1 family, member A1; *Bmi1*: B lymphoma Mo-MLV insertion region 1 homolog; cIAP-2: Cellular inhibitor of apoptosis 2; DOT1L: DOT1 like histone lysine methyl transferase; EGFR: epidermal growth factor receptor; EMT: epithelial mesenchymal transition; FGF2: fibroblast growth factor; FGFR2: fibroblast growth factor receptor 2; GLI1: glioma-associated oncogene; HDAC1: histone deacetylase 1; HDAC2: histone deacetylase 2; HNSCC: head and neck squamous cell carcinoma; KAT6A: Klysine acetyltransferase 6A; KAT6B: Klysine acetyltransferase 6B; KLF4: kruppel-like factor 4; NOTCH1: neurogenic locus notch homolog protein 1; Oct4: octamer-binding transcription factor, OSCC: oral squamous cell carcinoma; SDCBP: syndecan-binding protein; SIRT1: sirtuin 1; SMO: smoothened, frizzled class receptor; Sox2: sex-determining region Y [SRY]-box; Sox8: SRY-box transcription factor 8; TSPAN1: tetraspanin-1; ZEB1: Zinc finger E-box-binding homeobox 1XIAP = X-Linked Inhibitor of apoptosis.

Interestingly, when considering the CSC phenotype and plasticity in chemoresistant HNSCC tumor samples and cell lines, members of the regulator of embryonic stem cell *Sox* and *Oct4* are highlighted over the classical CD44, Bmi1, and even ALDH1 CSCs biomarkers. *Sox2* was associated with clinicopathological parameters of worse outcomes in HNSCC patients and a mediator of therapy resistance *in vitro*. Functionally, *Sox2* induced the expression of the anti-apoptotic protein Bcl-2 and enhanced resistance to apoptosis-inducing agents, including cisplatin^[108]^. Accordingly, Lee *et al.* found that *Sox2* overexpression was correlated with tumor recurrence and poor prognosis in HNSCC, contributing significantly to the acquisition of stem cell traits *in vitro*^[109]^. Ectopic expression of *Sox2* in HNSCC cells induced stemness by positive regulation of *Oct4* and *Nanog* and co-expression of CD44. In addition, endogenous levels of *Sox2* were significantly higher in ALDH1 high cells. At the same time, the downregulation of *Sox2* was followed by *Oct4* and *Nanog* down-regulation, decrease in stemness, invasion, EMT mediators, *in vivo* tumorigenicity, and frequency of CD44^+^ cells. Moreover, *Sox2* enhances the chemoresistance of CSCs to cisplatin, possibly by inhibiting ABCG2 expression and resistance to oxidative stress in CD44^+^ CD271^+^ CSCs in HNSCC^[109]^.

Xie *et al.* found that *Sox8* expression was positively associated with chemotherapeutic resistance, higher lymph node metastasis, advanced tumor stage, and shorter overall survival in HNSCC patients^[110]^. Also, the expression of *Sox8* in cisplatin-resistant HNSCC cell lines is responsible for orchestrating the acquisition of the CSC phenotype *via ABCG2*, *Sox2*, *Oct4*, and *Bmi1* expression but also resistance to therapy and activation of EMT and Wnt/β-catenin pathway, favoring tumor invasion and progression. These findings indicate that *Sox8* could be used as a biomarker and a possible target to eradicate the CSCs and increase tumor response to standard therapies toward HNSCC^[110]^.

Several *in vitro* studies investigating the relevance of CSCs on chemoresistance initially characterize the CSCs subpopulation based on the expression levels of CD44 and ALDH1. Nör *et al.* showed that treatment with low doses of cisplatin promotes *Bmi1* and *Oct44* expression and increases the CSCs fraction identified as CD44^high^ ALDH^high^, indicating that these cells are intrinsically resistant to treatment and can expand after therapy^[111]^. The study by Chen *et al.* elegantly confirmed that Bmi1^+^ CSCs are enriched *in vivo* after treatment with cisplatin, being able to reconstitute the tumor heterogeneity and are the main responsible for recurrence^[112]^. Kulsum *et al.* found that HNSCC cell lines resistant to cisplatin and 5-FU showed enrichment of CD44^+^ ALDH1^+^ subpopulation, stemness, expression of *ABCG2*, *Sox2*, *Nanog*, *Oct4*, and *NOTCH1* genes, and G0/G1 or S phase arrest^[113]^. One of the mechanisms by which the CD44^high^/ALDH1^high^ cells become resistant may be the upregulation of the DOT1L and monomethyl l-H3K79 that lead to miR-10 activation, resulting in cytoskeleton remodeling *via* RhoC and upregulation of prosurvival molecules such as cIAP-2 and XIAP^[114]^. Another mechanism associated with cisplatin resistance of CD44^high^/ALDH1^high^ is the secretion of FGF2. Most importantly, cisplatin combined with FGFR2 inhibition decreased the percentage of CD44^high^/ALDH1^high^, and no CSCs enrichment was noticed after cisplatin exposure, indicating that blocking FGFR is an attractive target to eliminate the CSCs in HNSCC^[115]^.

CD44 is frequently associated with other potential markers of CSC aiming for efficient enrichment of this subpopulation within HNSCC cell lines and tissues. Galbiatti-Dias *et al.* identified the CSC profile of HNSCC cell lines as CD44^high^ CD133^high^ CD117^high^ profile^[116]^. This CSCs subpopulation demonstrated higher migration capacity and more resistance to Paclitaxel chemotherapy, in addition to an up-regulation of CD44 and down-regulation of *EGFR* transcripts in the HN13 oral cancer cell line^[116]^. Elkashty *et al.* combined the positivity of CD44 to CD271 (p75NTR), a described marker of CSC in many tumors^[117]^, to isolate an enriched subpopulation of CSCs, followed by their characterization *in vitro*, *in vivo*, and HNSCC tissue samples. The authors found that CD44^+^ CD271^+^ cells exhibited higher cell proliferation, sphere/colony formation, chemoresistance to cisplatin and 5-FU, and radioresistance, upregulation of CSCs-related genes (*Sox2*, *Oct4*, *Bmi1*, *Smo*, and *GLI1*), and *in vivo* tumorigenicity^[117]^. These combined cell markers also showed increased expression in patients with advanced disease.

A study from Oh *et al.* demonstrated that CD44^+^ cells derived from primary HNSCC had increased expression of ABCG2 and enriched side population^[118]^. Yu *et al.* found that side population cells characterized by the CD133^+^ phenotype show elevated chemoresistance and ABCG2 expression, which was abrogated by combining cisplatin with CD133-targeted therapy^[119]^. Moreover, *Snail* is overexpressed in CD44^+^ CSCs and associated with cisplatin resistance and high expression of ABC transporters^[120]^. Interestingly, the percentage of *Oct4* positive cells increases significantly after treatment with 5-FU, cisplatin, and paclitaxel^[121]^, and increased expression of ABCC6 was associated with increased resistance to cisplatin in *Oct4* overexpressing cells, indicating that this poor explored ABC transporter may be relevant to resistance acquisition in HNSCC^[122]^. Thus, constitutive or acquisition of stem cell and EMT-associated genes are involved with the up-regulation of drug transporters pumps and multi-drug resistance.

The process of EMT is tightly linked with the CSC’s biology and chemoresistance in HNSCC. CSCs keep their EMT phenotype until depositing in the distant sites of metastasis (a migratory phenotype), where they change their phenotype toward attaining a MET morphology to proliferate rapidly, causing tumor outgrowth (a proliferative phenotype)^[123]^. This rapid cellular proliferation leads to hypoxia in the nearby milieu, thereby exacerbating tumor resistance to therapy^[124]^. Masui *et al.* observed that the CSC-like phenotype is induced after *Snail*-overexpression and is associated with increased CD44^+^/ALDH^+^ in HNSCC cell lines^[125]^. The EMT and CSC phenotype acquisition in *Snail* overexpressing cells also decreased chemosensitivity. Similarly, Ota *et al.* demonstrated that *Snail*-induced EMT was associated with increased stemness, inducing *in vivo* cancer invasive progression and enhancement of chemoresistance^[126]^. A recent study from Oliveira *et al.* demonstrated that the CSC subpopulation and activation of the EMT program, characterized by down-regulation of E-cadherin and up-regulation of vimentin, mainly *via* association of epigenetic regulators and ZEB1, is involved with resistance to cisplatin in HNSCC cell lines^[96]^.

It is worth mentioning that the ability of tumor cells to dynamically adapt to signals provided by the tumor microenvironment and/or induced in response to therapy is obtained by the property of cell plasticity at different stages of tumor progression. Cancer cell plasticity reflects genetic and epigenetic alterations in tumor cells, promoting phenotypical diversity and contributing to intra-tumor heterogeneity^[127]^. EMT and CSCs states are the two most studied axes of tumor cell plasticity and are often tacitly assumed to be synonymous^[128]^. This is because both cell plasticity axes appear to drive one another *in silico*, *in vitro*, and *in vivo* studies^[129]^. Notably, both mathematical modeling studies and experimental observations have reported that EMT is also not a unidirectional process since there are one or more hybrid epithelial/ mesenchymal (E/M) states between the two extremes of pure epithelial or pure mesenchymal phenotypes^[130,131]^ during EMT. For this reason, the term Epithelial-Mesenchymal Plasticity (EMP) has been used as a more accurate description of the process.

The same is true for CSC since there may be subsets of CSCs defined as epithelial, mesenchymal, and hybrid E/M (E-CSCs, M-CSCs, H-CSCs)^[132,133]^. According to Sahoo *et al.* 2022^[128]^, the emerging evidence points to EMT and stemness being semi-independent axes, i.e., not every cell undergoing EMT may acquire stemness and not every cell switching to be a CSC is mandated to show one or more features of EMT. These authors recently proposed a mathematical model to understand the interconnectivity between the EMP and stemness axes aiming to elucidate the critical cellular processes driving metastasis. This model allows many possible couplings between EMP and stemness, showing that all phenotypes - epithelial, mesenchymal, and hybrid E/M - have the potential to be stem-like; however, this potential is likely to be maximum for hybrid E/M cells^[128]^. On the other hand, tumor cells exhibiting an amoeboid phenotype belong to the utterly mesenchymal end of the EMP spectrum but show high stemness and metastatic potential^[134]^. So, many stem cell phenotypes exist across the EMP spectrum that would only be identified based on single-cell RNA sequencing approaches^[128]^.

Garcia-Mayea *et al.* showed that CSCs isolated by sphere formation in non-adherent conditions were more resistant to cisplatin and 5-FU, possibly due to the increased levels of LC3II/I, indicating that autophagy may be involved in within-drug resistance of CSCs^[135]^. Recently, using the same CSC model, these authors identified by RNAseq the *TSPAN1* (Tetraspanin 1) gene as an essential modulator of chemoresistance in HNSCC^[136]^. Blocking *TSPAN1* demonstrated encouraging *in vivo* results, leading to impaired tumor growth, EMT acquisition, and metastasis spreading. Another possible target to eliminate HNSCC CSCs and cisplatin resistance is the SDCBP (Syndecan-binding protein), a central contributor in different phases of the metastasis cascade^[137,138]^. Upon fibronectin and extracellular molecule engagement, SDCBP, as an adaptor protein, interacts with Src and forms a stable complex with FAK in the cellular membrane leading to long-term Src activation. As a result, downstream target signaling pathways such as NF-kB and TGF-β are activated, promoting EMT, tumor migration, invasion, metastasis, and cisplatin resistance^[138]^. Lee *et al.* showed that Wnt/β-catenin signaling is activated in CSCs, and β-catenin overexpression drives the acquisition of CSCs properties as self-renewal, stem cell marker expression, including *Oct4*, and chemoresistance^[139]^. In hypoxic conditions, HIF-1α activates NOTCH1, which is responsible for stemness, EMT activation, and resistance to cisplatin in CD44^+^ cells^[140]^.

All these exposed findings reveal how broad and complex the process of resistance to the chemotherapeutics available today for treatment could be. It also guides us to seek new and innovative drugs focused on CSCs, such as targeted therapy and immunotherapy, for better treatment and prognosis of HNSCC patients. Notably, the plasticity of CSCs must also be considered since their dynamic phenotype switch may be responsible for different levels of resistance even in the same tumor type. As pointed out by Biddle & Marles^[141]^, an effective biomarker should be precise in correlating the presence of phenotypically plastic CSCs with tumor aggressiveness and therapeutic resistance. It would allow more accurate clinical decisions, such as neck dissection and chemotherapy regimens in HNSCC. More recent evidence highlights some meaningful advances, for example, monoclonal antibody therapy anti-CD44v6 and other markers related to EMT signaling pathways activation, such as the Notch, WNT, and ERK/ MAPK pathways. Although, in terms of clinical safety, targeting CSC-specific processes is not well established yet.

## CONCLUDING REMARKS

The presence of CSCs in HNSCC and other solid tumors is associated with tumor heterogeneity and resistance to standard therapies. Target CSCs therapy is very challenging as these cells are a dynamic and plastic population capable of switching between different phenotypes and activation states according to the stimuli provided by the TME. As a result, the frequency of CSCs and their spatial localization in the primary tumor and metastatic foci may be variable, leading to different levels of tumor resistance after treatment. Many studies demonstrated that after radio and chemotherapy, CSCs are enriched and guide tumor recurrence and progression.

In this scenario, it is mandatory to characterize the CSCs and their mechanisms of interactions with the TME in HNSCC to better design targeted therapies that efficiently eliminate these cells in combination with standard treatment and/or immunotherapy. Disrupting the TME can lead to hypoxia inhibition and disturb the CSC niche, facilitating CSCs sensitization to chemotherapy. Moreover, CSCs interaction with different cell types in the TME may be impaired, facilitating its elimination and response to standard treatment. It is essential to highlight that CSCs have an efficient drug efflux machinery that should be considered as possible targets to improve drug accumulation within this subpopulation of tumor cells. Targeting signaling pathways involved with acquiring stemness, such as the Wnt/β-catenin, FGF, and NOTCH1 in HNSCC, may also be an attractive strategy to eliminate the CSCs and drug resistance. Taken together, CSCs are a relevant target to achieve control of disease and treatment response in HNSCC as they represent significant drivers of tumor resistance. Future studies, especially those using cutting-edge methodologies such as scRNAseq, will help to identify new CSCs targets and cellular interactions that can be used to develop new multi-faceted adjuvant therapies.

## DECLARATIONS

### Copyright

© The Author(s) 2023.
